# Traumatic Brain Injury: An Age-Dependent View of Post-Traumatic Neuroinflammation and Its Treatment

**DOI:** 10.3390/pharmaceutics13101624

**Published:** 2021-10-06

**Authors:** Clément Delage, Toufik Taib, Célia Mamma, Dominique Lerouet, Valérie C. Besson

**Affiliations:** 1 Université de Paris, Inserm UMR-S 1144—Optimisation Thérapeutique en Neuropsychopharmacologie, Faculté de Pharmacie de Paris, 4 Avenue de l’Observatoire, 75006 Paris, France; dominique.lerouet@u-paris.fr (D.L.); valerie.besson@u-paris.fr (V.C.B.); 2 Université Paris Descartes, EA4475—Pharmacologie de la Circulation Cérébrale, Faculté de Pharmacie de Paris, 4 Avenue de l’Observatoire, 75006 Paris, France; toufikpharma@gmail.com (T.T.); cyliamam@gmail.com (C.M.)

**Keywords:** neuroinflammation, traumatic brain injury, preclinical, neonates, juvenile, adolescent, aged, preclinical, neuroprotection

## Abstract

Traumatic brain injury (TBI) is a leading cause of death and disability all over the world. TBI leads to (1) an inflammatory response, (2) white matter injuries and (3) neurodegenerative pathologies in the long term. In humans, TBI occurs most often in children and adolescents or in the elderly, and it is well known that immune responses and the neuroregenerative capacities of the brain, among other factors, vary over a lifetime. Thus, age-at-injury can influence the consequences of TBI. Furthermore, age-at-injury also influences the pharmacological effects of drugs. However, the post-TBI inflammatory, neuronal and functional consequences have been mostly studied in experimental young adult animal models. The specificity and the mechanisms underlying the consequences of TBI and pharmacological responses are poorly understood in extreme ages. In this review, we detail the variations of these age-dependent inflammatory responses and consequences after TBI, from an experimental point of view. We investigate the evolution of microglial, astrocyte and other immune cells responses, and the consequences in terms of neuronal death and functional deficits in neonates, juvenile, adolescent and aged male animals, following a single TBI. We also describe the pharmacological responses to anti-inflammatory or neuroprotective agents, highlighting the need for an age-specific approach to the development of therapies of TBI.

## 1. Traumatic Brain Injury

The aim of this review is to present the principal elements of traumatic brain injury (TBI), post-traumatic neuroinflammation, and to describe its evolution and consequences depending on age (neonates, juvenile, adolescent and aged), based on preclinical data.

### 1.1. Definition

TBI is defined as an alteration in brain function, or other evidence of brain pathology, caused by an external force [1]. Characterized by a great heterogeneity in terms of etiology, physiopathology, severity and symptomatology, TBI is considered the most complex disease in our most complex organ [2]. TBI is now also considered a chronic condition, as it can progress not only for hours or days but also for years. Depending on the injured brain regions and the extent of these lesions and symptoms, TBI may be classified as mild, moderate or severe. Mild TBI represents 80 to 90% of all TBI [3,4]. The proportion of severe TBI is lower, but is characterized by a higher mortality rate, of 30 to 40%.

### 1.2. Epidemiology

More than 50 million TBI cases occur each year worldwide and about half of the world population will undergo, at least, one TBI in their lifetime [4]. The two main populations sustaining TBI are people aged <4 years and those aged >75 years. People between 5 and 25 years are also more likely to sustain TBI, but to a lesser extent. TBI may originate from road traffic incidents, falls, sports, terrorism and military conflicts. A recent systematic review and meta-analyses of articles describing the epidemiology of TBI in 16 European countries showed that the first two causes are the most frequent, with falls being reported more frequently than motor vehicle accidents [5]. However, within the studies that mainly focus on more severe TBI, the latter remain dominant as a cause of injury. Moreover, a correlation was found between the cause of injury and age, with falls being most common in children and elderly subpopulations, while road traffic incidents are the most frequent in young adults. TBI causes vary greatly between countries and regions. In low- and middle-income countries, the rising burden of TBI due to the increase of road traffic incidents mainly affects youths, while in high-income countries the changing epidemiology of TBI is related to a high and increasing incidence in infants and elderly people. Lastly, sports, such as rugby and boxing, and military conflicts also increase the number of TBIs [5,6].

### 1.3. Neuroinflammation after TBI in Young Adults

TBI leads to primary lesions resulting directly from an impact and caused by mechanical forces. These physical injuries will trigger physiopathological and biochemical cascades, leading to secondary lesions [7], which tend to exacerbate the initial damages in the hours, days, months or even years after the impact [8]. Among these secondary lesions, the “sterile” (as it is not caused by pathogens) inflammatory response is called neuroinflammation. Neuroinflammation is a complex, nonspecific and coordinated response that appears during the acute phase of TBI and can last for years. Neuroinflammation is characterized by (1) the activation of glial cells by cellular membrane disruption components like the damage associated molecular patterns (DAMPs) and/or mitochondrial dysfunction components, and/or blood components leakage from the altered blood-brain barrier (BBB); (2) the release of pro- and anti-inflammatory cytokines; (3) the infiltration of blood cells through BBB, sometimes injured by the impact [9].

#### 1.3.1. Immune Glial Cells

Activated microglia and astrocytes phagocyte cellular debris and modulate the other immune cells responses. In an inflammatory context, these cells undergo morphological changes and form a glial scar, constituting a physical barrier that isolates the lesion area from other healthy brain structures. Glial cells can be activated directly by brain lesions, but also by perivascular glial cells and infiltrating immune cells. They also can be activated by their own secreted factors in an autocrine and paracrine way, which will then amplify and self-sustain the inflammatory response [10,11].

##### Microglia

Microglial activation

Microglia are the specific resident macrophage of the central nervous system (CNS) with phagocytosis and antigen presentation capabilities. Microglia are the most abundant mononuclear phagocyte in the CNS and accounts for about 10% of the total CNS cell population in adults [12]. Under physiological conditions, microglia are ramified and very dynamic, constantly ensuring the homeostasis of its environment. Its ramifications monitor the cerebral parenchyma to remove tissue debris, excess metabolites, or any other component that may disrupt CNS homeostasis by phagocytosis. In addition, microglia are essential for neuronal development and function. It not only participates in the control of cell death and the elimination of defective synapses, but also contributes to neurogenesis, transmission and synaptic plasticity. In the absence of brain injury, microglia are maintained in the “surveilling” state through “off” signals mainly from neurons [13].

Depending on stimuli to which microglia are subjected, they will differentiate into different activation state, leading to microglial phenotypes or the notion of microglial polarization. As microglial activation is outside the scope of this review and has already been widely described in exhaustive reviews [13,14], we will only briefly underline the different microglial phenotypes. Activated microglia are commonly categorized by two phenotypes, M1, considered pro-inflammatory, and M2, considered anti-inflammatory. The different microglial phenotypes work together to control inflammation, remove debris and promote tissue repair and remodeling. The action of pro-inflammatory molecules on microglia directs them towards the pro-inflammatory M1 phenotype, which produces pro-inflammatory cytokines (TNFα, IL-1β, IL-6, -12, -18, -23, IFNγ) and chemokines (CCL2, 5 and 20, CXCL1, 9 and 10) as well as reactive oxygen species (ROS). In contrast, in response to anti-inflammatory cytokines (IL-4, -10 and -13, TGFβ), microglia adopt an alternative activation phenotype designated M2. This phenotype has anti-inflammatory properties and participates in tissue repair and regeneration. M2 subphenotypes have also been described, with an “alternative” M2a, an “intermediate” M2b and a “deactivated” M2c phenotype. However, as almost no study explored M2 subtypes expression in TBI other than in young adult rodents, we will not detail their role in this review [13]. Although microglial polarization is well described in in vitro models, the proof of existence of two distinct populations M1 and M2 in vivo is currently under debate [15]. Faced with very complex tissue signals, microglia can also adopt a mixed phenotype called “Mtrans”, expressing both M1 and M2 markers [16]. Indeed, a continuum of microglial activation, expressing different microglial states markers, is observed after a TBI [14].

Microglial activation consequences in vitro

In vitro, microglial phenotypes M1 and M2 can be induced by the application of respectively pro-inflammatory and anti-inflammatory cytokines on primary culture of microglial cells.

The M1 phenotype inhibits the differentiation of neural progenitor stem cell (NPC) into mature oligodendrocytes [17], exacerbates neuronal and oligodendrocyte death following oxygen and glucose privation [18,19,20,21] and secretes pro-inflammatory cytokines (such as TNFα, IL-1β, -2 and IFNγ) which induce apoptosis in mature oligodendrocytes [22].

Conversely, the M2 microglia phenotype exerts its neuroprotective activity by promoting neurogenesis through the secretion of brain-derived neurotrophic factor (BDNF), nerve growth factor (NGF) and basic fibroblast growth factor (FGF-2) [16], and neural-progenitors differentiation [17]. M2 phenotype has also a protective effect on oligodendrocytes cell death induced by oxygen and glucose deprivation [21].

Moreover, it has been shown that myelin debris resulting from neuronal death also inhibit differentiation of oligodendrocyte progenitor cells [23,24] and activate microglia [25], in a pro-inflammatory [26] and neurotoxic [27] phenotype. Myelin debris inhibit neuronal growth and axonal regeneration [28]. Myelin debris do not seem to have an exclusively deleterious effect, as macrophages phagocytizing myelin debris present rather anti-inflammatory properties [29]. However, only a few studies have investigated the implications of myelin debris in post-TBI neuroinflammation. 

Microglial activation in vivo in young adult

In vivo, in young adults, microglial activation occurs from 4 h [30] to one year post-TBI [31]. Different subsets of microglia increase during the acute and chronic phases after TBI. The M1 phenotype appears early, from the first hours post-TBI [32]. The M2 phenotype appears later [19] and transiently [16,19,32,33,34,35]. Thus, after a M1/M2 cohabitation phase with the presence of a mixed activated population, up to 5 weeks post-TBI [10,36], the M1 phenotype eventually predominates [33] and remains the only active phenotype [19].

Additionally, some studies showed the presence of “Mtrans” microglia coexpressing M1 markers such as TGFβ, CD16 and CD32, and M2 markers such as Arginase1 and NOS2 from 1 to 7 days post-TBI [16,35].

##### Astrocytes

Located between endothelial cells and neurons, astrocytes help maintain the BBB integrity by forming astrocyte feet surrounding endothelial cells [37]. They also participate in neuronal homeostasis by modulating neurotransmitter concentrations such as glutamate and promoting the formation and function of synapses [38]. Astrocytic activation following brain damage is designated by the term “astrogliosis” [39]. Once activated, astrocytes proliferate, change their morphology with hypertrophy of cell body and processes, and produce inflammatory mediators and trophic factors [40]. They also migrate to the lesion site and form the glial scar, which prevents the diffusion of pro-inflammatory and cytotoxic molecules to surrounding healthy tissues and thus limits the extension of the inflammatory process outside the lesion area [38].

As for microglial activation, astrogliosis has been found to be complex, since both beneficial and deleterious effects have been attributed to them [41]. Indeed, neuroinflammation induces two different types of reactive astrocytes that can be termed “A1” and “A2” based on M1 and M2 macrophages nomenclature. The A1 phenotype loses most normal astrocyte functions but gains neurotoxic properties, rapidly killing neurons and mature differentiated oligodendrocytes. Oppositely, the A2 phenotype appears neuroprotective [42]. If the A1 phenotype has been reported following TBI [43], its effects following TBI have nonetheless not yet been studied. However, as for the microglial phenotype denomination, recent studies suggest that there is no reactive astrocytic polarization into simple binary phenotypes. Reactive astrocytes may adopt multiple states depending on context, with only a fraction of common changes between different states [44].

In vivo, in young adults animals, astrogliosis appears in the hours following the impact and can last for 14 days to 60 days [30,45,46,47].

#### 1.3.2. Cytokines

Cytokines can be secreted by all cellular actors of inflammation, as well as by neurons, but microglia remains their main source in the CNS [48]. In addition to their role in inflammation, cytokines are essential for CNS development and function [45]. Cytokines, and their role, have already been widely described in well-documented reviews [49,50,51] so we will only briefly describe the main different cytokines implicated in neuroinflammation.

Pro-inflammatory cytokines that initiate inflammation include IL-1β, -6 and -18, TNFα and IFNγ. These cytokines are first released by glial cells after tissue injury and:

- promote the activation and proliferation of astrocytes and microglia;
- contribute to the formation of edema and neuronal death and inhibit neuronal regeneration;
- potentiate the permeability of the BBB and increase the expression of cell adhesion molecules and chemokines to recruit peripheral immune cells;
- stimulate the production of other pro-inflammatory cytokines, or enzymes such as cyclooxygenase-2 or phospholipase A2.

The effects of pro-inflammatory cytokines are not so settled and unilateral, and contribute to the complexity of the neuroinflammation. Several studies reported detrimental effects of inhibiting pro-inflammatory cytokines, meaning they could confer beneficial and long-term protective effects [45].

Anti-inflammatory cytokines, such as IL-4, -10, -13, and TGFβ, appear after the pro-inflammatory ones to modulate and reduce inflammation by mechanisms that are still not fully elucidated [8]. 

Cytokine expression in vivo following TBI in young adult animals has already been widely studied and reviewed [52]. Briefly, overall cytokine expression increases from 1 h following TBI and peaks between 2 and 24 h following injury [52]. IL-1β expression increases within 1 h and peaks between 12 and 24 h following TBI [53,54,55,56,57]. TNFα expression increases within 1 h and peaks between 4 and 8 h following TBI [58,59,60,61,62]. IL-6 expression increases at 1 h and peaks between 2 and 8 h following TBI [58,59,63,64,65,66,67]. IL-10 expression increases rapidly and peaks between 4 and 20 h following TBI [68]. 

#### 1.3.3. Peripheral Immune Cell Infiltration

Some cytokines—chemokines—are capable of regulating the activation and migration of leukocytes through the BBB, already potentially altered by the impact of TBI [9]. Leukocytes, thus recruited by the chemotactic molecules, roll along the endothelium, adhere to the latter through adhesion molecules (ICAM-1), then pass through the BBB via the intercellular junctions [69]. In brain parenchyma, they reinforce and stimulate the inflammatory activity of CNS cells, and contribute to the recruitment of new peripheral immune cells. It contributes to cytotoxicity, neuronal death, glial activation and inflammatory mediators production that disrupt the BBB permeability [9,70]. In vivo in young adults animals, leukocytes infiltration appears to be very fast, from a few hours after the impact [70,71], but the number of macrophages begins to decrease after only a few days TBI [33].

### 1.4. Neuroinflammation Consequences in Young Adults

Although neuroinflammation is essential for exogenous pathogens’ elimination, tissue repair and neuroregeneration after TBI, this “double-edged sword” inflammatory response [72] amplifies post-TBI primary and secondary lesions, especially neuronal death and white matter injuries (WMI) [8,73]. Due to the spatio-temporal correlation between (1) microglial activation and axonal lesions [74], (2) the early increase of IL-1β brain expression and severity of the TBI [45,48,49], and (3) the frequent association between chronic microglial activation and white and gray matter degeneration [31,75,76,77], chronic neuroinflammation seems to contribute to the long-term neurodegeneration following TBI [31,78].

Cellular death and white matter injuries

It has been demonstrated that the corpus callosum is altered, with white matter thinning and decreased myelin staining, at the short and long terms in rodent models of TBI, [79,80,81,82]. An oligodendrocyte death has been observed up to 21 days after TBI [83]. As oligodendrocytes are responsible for myelin synthesis, their destruction induces demyelination that will slow nerve conduction and favor axonal degeneration and WMI [31,76,84,85]. Oligodendrocyte death could be due to the M1 microglial phenotype. This hypothesis is supported by the correlation between the number of microglia and the severity of demyelination and axonal damages [19], and the preservation of white matter integrity when decreasing M1 activation [21]. The M1 microglial phenotype would thus be responsible of myelin decrease at short-(2 days) [85], mid-(14 days) [32] and long-term (2 months) post-injury [80]. However, oligodendrocytes are highly vulnerable to oxidative stress, excitotoxicity and hypoxia consecutive to TBI primary brain damages, which promotes their apoptosis [22,86,87]. Thus, M1 microglia potentiate an already present post-TBI oligodendrocyte death and neurodegeneration due to the TBI biochemical content [13]. However, another hypothesis to the decrease of oligodendrocytes could be proposed, since the death of oligodendrocytes after a TBI is associated with demyelination [32]. The destruction of neuronal axons results in the destruction of myelin, which could be the cause of oligodendrocyte death.

In a TBI context, microglial activation is associated to the presence of myelin debris [74,85,88]. As activated microglia aggravate oligodendrocytes death and demyelination [32] it could induce myelin debris production, suggesting a vicious circle of neuroinflammatory amplifying and thus maintaining of the M1 phenotype [26] and its deleterious neuroinflammation [25,27,89,90]. Furthermore, as myelin is rich in lipids, WMI such as demyelination may be associated with an altered lipid profile near the lesion [91,92], in other brain regions [93,94] and in blood [95]. This has been confirmed by a lipidomic study, revealing modifications in the lipid profile of corpus callosum following a TBI [96]. A positive correlation was also observed between the increase in circulating sphingolipids and lesion volume, suggesting a possible use of the plasma level of sphingolipids as a biomarker for the diagnosis of brain lesions [97]. 

Demyelination is, however, not a definitive phenomenon, as remyelination and proliferation of oligodendrocyte progenitor cells has been observed following TBI [22,83,85,98], whose onset coincides with the transition from the M1 phenotype to the M2 phenotype [99]. Consistent with the in vitro effects of the M2 phenotype previously described, its increase is associated with preservation of the integrity of the white matter, decreases in axon demyelination and improvement in nerve conduction [21]. This phenomenon could explain why an undifferentiated total depletion of microglial phenotype does not always demonstrate protective effects [100,101].

Infiltrating immune cells also contribute to post-traumatic chronic inflammation and its functional consequences, since inhibition of chemotaxis, or polymorphonuclear neutrophils depletion induces a decrease in cytokine production, cerebral oedema, injury volume and tissue loss, microglia/macrophage activation, cellular death and neurobehavioral deficits [102,103,104].

Epileptogenesis

Post-traumatic epilepsy is a well-known complication of TBI, experimentally evidenced in rodents both at the short and long terms [105,106]. Recently, the occurrence of epileptic seizures has been linked to the presence of neuroinflammation. Experimental studies also showed that the development of these seizures depends on the severity of neuroinflammation [107].

Specifically, inflammatory mediators released by brain cells, or infiltrating peripheral immune cells, following TBI activate cognate receptors expressed by neurons and induce transcriptional and post- translational changes in glutamate and GABA receptors and in ion channels [107]. The oxidative stress, induced by TBI through the generation of reactive oxygen species by activated immune cells and suffering neurons, also induces neuronal excitability [108]. Finally, BBB lesion and astrocytic activation contribute to epileptogenesis through water homeostasis disruption [109].

Epileptic seizures, thus generated, induce the release of neuronal death and reactive oxygen species, inflammatory mediators, released by immune cells or activated vessels, and BBB alteration, which in turn favors the inflammatory process [107]. This vicious circle maintains and potentiates post-TBI neuroinflammation.

Functional deficits

The WMI induced by neuroinflammation is increasingly recognized as responsible for long-term disabilities consecutive to TBI. Experimentally, in young adults animals, the extent of WMI has been shown to correlate with the degree of functional deficits months after injury [110,111]. These long-term functional deficits can be evaluated in several different ways [112]. Thus, motor coordination deficits following TBI have been observed through various tests, such as balance beam [113], beam walking [114], or rotarod tests [115]. TBI also induces memory deficits, as acquisition and reference memory deficits have been evidenced by the Morris water maze test [116] or the radial arm maze test [117]. Exploration behavior is also impaired after TBI, as evidenced by the novel object recognition test [118]. Concerning emotional functions, an alteration in anxiety-like behavior through the open-field test [119] and a depression-like behavior, through the forced swim test, have been observed [120].

### 1.5. Therapeutic Strategies

The medical management of TBI is only symptomatic. It mainly consists in the treatment of intracranial hypertension and convulsive status epilepticus, the maintenance and cessation of sedation and analgesia in patients [121]. To date, no therapeutic strategy is available and used to prevent the onset and spread of secondary lesions in humans.

Several pharmacological strategies have been tested in pre-clinical studies. They aim of reducing the progression of secondary brain lesions by administering anti-inflammatory or neuroprotective agents before or after TBI. Some therapeutic strategies also aim to inhibit the post-TBI epileptogenesis process to avoid the potentiation of neuroinflammation and the consequences of epileptic seizure. Thus, therapeutic strategies are more or less effective depending on the agent tested, the schedule of administration and the experimental model used, and have been the subject of well-documented reviews [122,123]. To date, none of them have yet passed the clinical development stage and been proven effective in humans [124].

Recently, it has appeared that the extent of the consequences of TBI depends not only on the severity of the mechanical impact, but also on patient-specific factors such as age [125] or sex [126]. Specifically, different microglial subtypes have been described as depending on age [127,128], presenting different characteristics and functions. Thus, as it has been suggested by some experimental studies [129], we could assume that the variation in the consequences of TBI due to age might depend on the variability of the inflammatory response. As Simon et al. highlighted in their very exhaustive review on post-TBI neuroinflammation, “*there is a need to define the inflammatory phenotypes of our patients on the basis of injury characteristics such as patient age, sex, genetic predisposition, presence or absence of secondary insults, and serum, CSF and/or imaging biomarkers*” [130]. 

**Figure 1:**
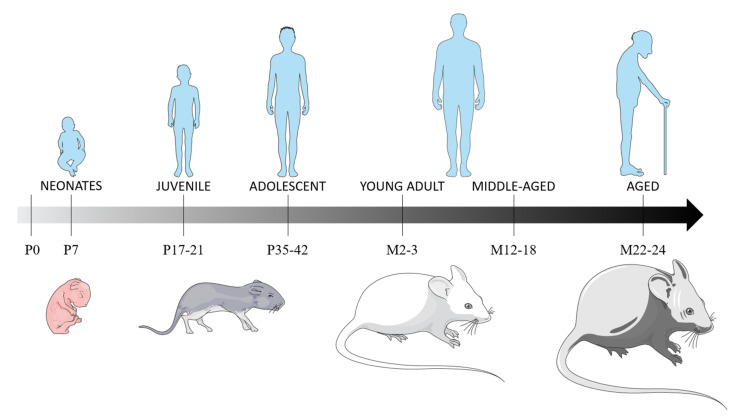
Rats’ and mice’ age correspondence with humans, based on brain and metabolism development, and sexual maturation. The brain developmental period occurs during the first three post-natal weeks, with the main brain growth-spurt period in the first post-natal week, a period of maximal neuronal proliferation, glial proliferation and the establishment of synaptic connections [132,133]. At P11, the neurological development in rodents is equivalent of a child below the age of 4 years [134]. Rodents can be considered juvenile at P17-21, based on equivalent synapse formation and γ-aminobutyric acid synthesis than in humans [135]. P35 rodents can be considered equivalent to preadolescent humans [136], as they will have developed 90% of their adult metabolic process [137] and as their sexual maturity is achieved at P60 [138]. *Abbreviations: P: postnatal day; M: postnatal months*.

The purpose of this review is to detail the variations of this age-dependent inflammatory response and its consequences, from an experimental point of view. TBI has been widely studied in young adults, which is usually used as the “reference” age to compare to other ages of TBI patients. Thus, we will not describe the evolution of the neuroinflammation response to TBI in young adults more than in §1.4 -neuroinflammation consequences in the young adult. We will focus on the consequences of TBI on neonates, juvenile/adolescent and old-aged animals, based on preclinical studies, as it corresponds to the age of the peak prevalence of TBI in humans (Figure 1). This review will only focus on single and not-repeated TBI, and will not investigate variation by sex, which is also suspected to influence the neuroinflammation response but has already been the subject well-documented review [126]. Since, to our knowledge only one study has been performed on both sexes in adolescents, we will only consider studies performed on males.

## 2. Post-Traumatic Neuroinflammation and Its Consequences In Vivo

Based on brain and metabolism development and sexual maturation, we considered rodents aged 7 postnatal days (P) as neonates, P17 to P21 as juvenile and P35 to P42 as adolescent. Rodents aged 12 to 18 months (M) were considered young adults and, those aged M22 to M24, as aged (Figure 1). We also included two studies of rabbit TBI models, as they are recent studies and as white matter development, microglial presence in the white matter tracts, and the pattern of brain growth is paralleled between rabbits and humans. Additionally, the rabbit brain has more anatomical similarity to the human brain than the lissencephalic rodent brain [131].

### 2.1. Neonates

Although infants are one of the most important populations that sustain TBI, only a few studies have explored its consequences in neonatal animals, and none have studied the long-term neurological and functional consequences thereof. All these studies are listed in Table 1. A closer look at pediatric TBI is needed, as the infantile period is characterized by several crucial developmental processes (brain growth, synaptogenesis, myelination…). In mice and rats, this developmental period occurs within the first three postnatal weeks [139]. In the literature, only P7 and P11 animals have been studied, as it is the period of maximal neuronal proliferation [139]. Processes at risk during this phase include glial proliferation and myelination, the explosive increase of dendritic complexity, the establishment of synaptic connections, and the reorganization of events [139] (Figure 1).

#### 2.1.1. Post-Traumatic Inflammatory Consequences 

The published data on the consequences of post-traumatic inflammatory on neonatal animals are summarized in Table 1.

Glial cells

Microglial activation is detected from 12 h post injury (hpi), peaks at 36–48 hpi, starts to decrease after 5 days post-injury (dpi) and is still present up to 35 dpi [139,142,144,145,150]. Isolated microglia and macrophages have only moderate changes in gene expression and express more markers of repair/regeneration and their immunomodulatory phenotypes [144].

Only one study reported an astrogliosis in neonatal animals, from 5 to 21 dpi [142]. According to the emerging role of astrocytes in microglial activation, this study also highlights the great need for more comprehensive studies on these cells.

Cytokines’ expression

A wide variation in cytokines’ and chemokines’ expression occurs from 1 to 5 dpi after TBI in neonates [143,144]. The pro-inflammatory/cytotoxic phenotype markers IL-1β and CCL3 show the greatest and most persistent increases in expression over time (>5 fold) in injured hemisphere. The anti-inflammatory or reparatory/regenerative cytokine IL-4 and the immunomodulatory cytokine IL-10 are also increased at 6, 14 and 24 hpi in the ipsilateral hemisphere. TNFα and IL-12 are the only markers that do not increase at these time points [144]. 

#### 2.1.2. Post-Traumatic Neuronal, Tissue and Functional Consequences

The published data on the post-traumatic neuronal, tissue and functional consequences in neonatal animals are summarized in Table 1.

Neuronal death

Neuronal mitochondrial activity and size increase early after TBI [145]. Neuronal death appears during hours following TBI [141] and is still present at 125 dpi [146]. Early neuronal death, from 0.5 to 24 hpi, is due to an excitotoxic mechanism and remains localized to the site of impact [140]. This sensibility to excitotoxic mechanisms might be explained by the poor antioxidant capacities of immature brains [139]. Late neuronal death is due to an apoptotic mechanism that begins at 6 hpi and persists for 5 dpi. Secondary apoptotic damages affect sites distant from the impact and appear to be more severe than primary excitotoxic damage [140,142,143,147,152].

Lesion size

Neonatal TBI induces tissue injuries in thalamus and hippocampus [144]; ventriculomegaly from 1 to 21 dpi [142,143,144] and atrophy of the sensorimotor cortex at 23 dpi [145].

Functional deficits

Few studies have examined the impacts of neonatal TBI in terms of functional deficits. Neonatal TBI induces cognitive deficits in adolescents, at 28 dpi, through an impairment in novel object recognition memory [151] along with an increase in the time spent in the open arms of the elevated-plus maze, suggestive of risk-taking behavior [148]. These functional deficits are still present in adulthood [146,151].

#### 2.1.3. Comparison with Other Ages 

To our knowledge, only three studies compared the consequences of TBI between neonates and older animals (Table 1), showing different consequences in terms of neuroinflammation, neuronal death and functional deficits in the neonates as compared with the adolescent/juvenile group.

Neonatal TBI induces a more important glial activation than juvenile/adolescent TBI [149]. Neonatal animals show an exacerbated neuronal loss and cortical and white matter atrophy following a TBI as compared with adolescent/juvenile animals. In older animals, damage remains limited to the impact site [140,147]. Neonatal animals also have more important acquisition deficits compared with adolescents/juveniles, suggesting that age-at-injury is a significant determinant of post-traumatic cognitive deficits [149].

#### 2.1.4. Anti-Inflammatory and Neuroprotective Therapeutic Strategies

One study tested the effect of minocycline, a second-generation tetracycline antibiotic with anti-inflammatory properties, in a neonatal TBI model [144]. The inhibition of microglial activation by minocycline [153] reduced injury severity at 1 day post-injury, reducing ventricular dilation and cell death. However, minocycline appears to have been only transiently neuroprotective in this model since it had no effect on injury severity at 5 days. Thus, the authors state that, unlike in adults, the role of activated microglia in injury mechanisms following TBI in the immature brain may not be only negative [144].

Another study used cromoglycate, an inhibitor of mast cells’ degranulation [154], in a neonatal TBI model [143]. Cromoglycate effectively decreased microglial activation markers. However, it did not reduce TBI-induced ventricular dilation, apoptosis or microglial number. This differs from the effects observed by the inhibition of mast cells in adult TBI [155]. Thus, the role of mast cells in TBI during development seems to be limited and not a viable target for therapies of TBI [143]. 

The findings of these only two studies that evaluated the effect of the administration of anti-inflammatory agents following a TBI in immature brain highlight the need for further specific therapeutic approach of preventing the consequences of TBI on newborns.

#### 2.1.5. Conclusions

Very few studies show interest in the consequences, even in the short term, of TBI in neonates. Especially, almost no studies compared the effect of TBI between neonates and older animals. This is mostly due to the impossibility of transposing the exact same injury model (localization and severity) from neonates to adults because of important differences in overall head, brain and skull anatomy. Thus, further works are needed to investigate mid- and long-term consequences, both on WMI and on neurobehavioral deficits, to explore the remote and persisting effects of TBI.

Nevertheless, these few studies indicate that neonates’ reaction to TBI differs from adults. Immature brain immune cells adopt a predominant anti-inflammatory and immunomodulatory phenotype following a TBI, which could explain their overall lower increase in cytokines expression compared with adult brains [144]. Additionally, unlike in adult TBI, the inhibition or depletion of microglia and macrophages is only transiently neuroprotective, as it reduces the lesion volume only at short term [144]. In the same way, mastocyte inhibition aggravates lesions in neonatal TBI, while appearing neuroprotective in adult TBI [143].

The poor antioxidant capacities of the immature brain make it more sensitive to excitotoxic mechanisms and aggravate post-TBI neuronal death [140,147]. Moreover, TBI in immature brains induces changes in the proteins involved in neuronal development (neuronal migration as well as axonal and dendritic growth and guidance) [139], suggesting long term functional deficits that should be evaluated. 

Finally, the scarce comparison of the consequences of TBI between different ages has nonetheless showed that neurobehavioral deficits decrease with brain maturity [149].

These studies highlight the difference in the consequences TBI for an immature brain compared with a mature one and suggest more severe consequences of TBI in the immature brain. They confirm the need for a specific approach for developing brain in the consequences of TBI.

### 2.2. Juvenile and Adolescent

Increasingly, studies are exploring the consequences of TBI in juvenile and adolescent populations, but very few, and very recently, show interest in neuroinflammation (Table 2 and Table 3). There is no precise definition of juvenile and adolescent ages for rodents and no guidelines for determining interspecies age equivalents. However, P12 and P13 rodents are considered equivalent to human newborns, based on synapse formation and γ-aminobutyric acid synthesis [135], and the literature considers juvenile rodent as P17-21. Additionally, the age of P17, in rats, is a critical myelination period, as it marks the peak of myelin basic protein (MBP) synthesis in the developing rat brain [156,157]. P35 rodents can be considered equivalent to preadolescent humans [136], as they have developed 90% of their adult metabolic process [137] and will have achieved sexual maturity at P60 [138] (Figure 1).

Yet, in this review, we considered literature’s qualifications for categorizing ages of juvenility and adolescence, which corresponded, in the following studies, with P17–P21 animals as juvenile and P35—P42 animals as adolescent.

#### 2.2.1. Post-Traumatic Inflammatory Consequences 

The published data on post-traumatic inflammatory consequences on juvenile and adolescent animals are summarized in Table 2.

Microglia

Microglial activation appears as early as 1 dpi and has been found up to 35 dpi [160,162,163,164,165,166,167,176]. It starts in structures close to the impact and extends to remote regions [166]. In rabbits at 7 dpi, microglia are activated in white matter tracts in the ipsilateral and, to a lesser extent, contralateral hemispheres [131].

Astrocytes

Astrocyte activation is absent before 1 dpi [138,158,165,166,170,171]. It begins at 1 dpi and lasts until 35 dpi [138,158,159,162,163,165,166,167,170,171,175,178], starting to spread to remote and contralateral regions from 7 dpi [159]. Astrogliosis is no longer present at 180 dpi [158]. Interestingly, a study reported microglial activation at 1 dpi but not astrocyte activation [164], suggesting that astrocytes do not contribute to microglial activation.

Other immune cells and cytokines expression

In the cerebellum, peripheral macrophages contribute to the inflammatory process at 1 and 21 dpi, contrarily to microglia, which are not increased at this time in this region [180].

Cytokine expression is widely modified from 6 hpi to 21 dpi [166,169,172,173,177,180]. Specifically, anti-inflammatory cytokines peak in the hours following the TBI, but decrease rapidly [169]. Pro-inflammatory cytokines peaks later, between 6 hpi and 3 dpi, and stay predominant until 21 dpi [166,169]. In the plasma, cytokine levels increase between 1 and 3 dpi, and return to normal levels at 7 dpi [180]. Overall, cytokines increased expression is correlated to the presence of activated microglia and astrocytes [166]. However, the precise expression patterns of the different cytokines and their relation to the activation of microglia and astrocyte phenotypes has not been studied, yet, in juvenile or adolescent TBI models. 

#### 2.2.2. Post-Traumatic Neuronal, Tissue and Functional Consequences

The published data on the post-traumatic neuronal, tissue and functional consequences on juvenile and adolescent animals are summarized in Table 3.

Neuronal death

Neuronal death appears from 6 hpi in the cortex [171] and spreads to the thalamus and contralateral hemisphere at 3 dpi [181] and to remote brain areas at 7 dpi [170,181]. This neuronal death persists into the long term [79,158,167,171,183,188] and is still visible at 6 mpi [185]. 

MBP fragmentation appears at 3 dpi [79] and persists until 60 dpi in the white matter tract, consistently, leading to decreased axonal conductance [79,158,162]. 

The neuronal loss, specifically through traumatic axonal injuries, is correlated in terms of localization to astrogliosis [170,171] and to activated microglia/macrophage [181,184], highlighting the implication of neuroinflammation in the neuronal loss.

Lesion size

This neuronal death leads to brain atrophy and WMI, visible from 7 to 60 dpi [79,131,158,163,171,185,188,190] and even at 6 mpi [173,185]. 

A magnetic resonance imaging analysis revealed the presence of blood in tissues and edema from 6 hpi to 3 dpi, even in mild juvenile TBI, with edema volume correlated to severity [186]. In rabbits, lesion volume increased from 16% at 3 dpi to 30% at 7 dpi, indicating ongoing secondary injuries at these time points [131].

Functional deficits

Correlated to the temporal evolution of lesion size, adolescent and juvenile animals show functional deficits, such as learning/memory [158,171,177,180,187,188,191], motor [79,158,172,180,183] and anxiety-like behavior deficits [79,158,162,180,188]. These deficits appear in the adolescence and last into adulthood [79,158,162,173,189]. Specifically, adolescent TBI increases the sensitivity to the rewarding effects of cocaine in adulthood [168]. A study also reported visual and optokinetic response deficits, correlated to microglia and astrocyte activation in the optical tract [167].

In rabbits, TBI resulted in cognitive deficits (T-maze and novel object recognition) at 9 and 19 dpi (P14 and P24) [131].

#### 2.2.3. Comparison with Young Adult TBI

Compared with young adult TBI, the immune cell infiltration, microglial activation, and phagocytosis of neuronal proteins is different in adolescent TBI (Table 2). Specifically, compared with young adults:

- microglial activation increases earlier and is stronger, and persist longer in adolescent TBI [161,168];
- astrocyte activation occurs earlier and is stronger in adolescent TBI [161];
- peripheral immune transcriptomic profiles differ in juvenile TBI [192];
- monocytes infiltration persists longer in adolescent TBI [168].

The post-traumatic neurological and functional consequences following adolescent TBI are more important than the consequences following adult TBI (Table 3). Specifically, compared with adults:

- dopamine system–related genes are altered [168];
- synaptic arborization complexity and spine density are reduced in the nucleus accumbens [168];
- functional deficits are similar [182], except for memory deficits, more important in adolescent TBI [184];
- mortality rate is higher following adolescent TBI [182,184].

#### 2.2.4. Anti-Inflammatory and Neuroprotective Therapeutic Strategies

Several studies evaluated the effect of anti-inflammatory or neuroprotective strategies in juvenile TBI model.

The anti-inflammatory approaches showed interesting results. The anti-inflammatory properties of docosahexaenoic acid decrease neuroinflammation and neuronal death in adult TBI models [193]. Similarly, in juvenile models, several studies showed that docosahexaenoic acid decreases microglial activation, macrophage infiltration, brain edema, oxidative stress, lesion volume, and short- and long-term neurological deficits following TBI [172,173,175]. Similarly, minocycline reduces microglial activation, attenuates neurodegeneration and increases neuronal survival following juvenile TBI [174], as in adult models [194]. Finally, dexamethasone, a steroidal anti-inflammatory molecule that has been shown to both decrease the post-TBI inflammatory response and promote neuronal plasticity, attenuated the cocaine place preference of injured juvenile animals [165].

Neuroprotective therapies are also effective in reducing the consequences of TBI in juvenile animals. The administration of isoflurane results in a decrease in microglial activation, neuronal death and cognitive and sensorimotor deficits [164]. These results are consistent with those found in adults [195]. However, the results of neuroprotective strategies do not seem to be always as effective in juvenile animals as it is in adult animals. Indeed, nicotinamide administration in juvenile TBI decreases microglial activation and neuronal loss [179], but does not reduce the lesion volume nor improve behavioral outcomes, as it does in adults [196]. This highlights the age-dependent nature of the response to pharmacological strategies and the need for more studies evaluating specific anti-inflammatory and neuroprotective approaches in juvenile models.

#### 2.2.5. Conclusions

Adolescent and juvenile TBI have been more studied than the two other specific ages of interest in this review. However, only a few have studied the long-term consequences of TBI, or directly compared juvenile or adolescent TBI to adult TBI.

As in adults, adolescent/juvenile TBI induces (1) glial activation and neuroinflammation leading to (2) neuronal death and WMI and to (3) functional deficits. Indeed, the administration of anti-inflammatory substances like dexamethasone, minocycline or docosahexaenoic acid decreases microglial and astrocyte activation [165,173,174,175,179] and peripheral immune cell recruitment [165]. These anti-inflammatory strategies also decrease WMI [173,174] and functional deficits [165,173,174,175]. These elements confirm the implication of neuroinflammation, directly or not, in WMI and functional deficits. However, minocycline did not have any effect on lesion volume, and although it promoted motor recovery, it worsened acquisition impairments [174], suggesting a more complex role of neuroinflammation than being only deleterious. 

The few studies that showed interest in the long-term consequences of TBI showed that functional deficits last for months after injury, into adulthood [79,162], especially cognitive deficits [158,185]. Mice that sustained TBI during adolescence also showed impaired social behavior [189] and increased sensitivity to the rewarding effects of cocaine in adulthood [168]. It highlights the lifelong consequences of an adolescent TBI. These findings are essential for human research in TBI, and the neurobehavioral consequences thereof should be the subject of further long-term study.

Comparative studies have highlighted different patterns of glial activation and neuroinflammation processed as compared with older animals [161,168,192]. They also reported variable consequences on brain lesion, neurodegeneration and neurobehavioral deficit [182,184]. Finally, in a juvenile rat TBI model, nicotinamide did not significantly improve behavioral impairments, conversely to older rat models, suggesting a different pathological pattern between juvenile and older adult TBI [179]. 

These studies highlight the need of (1) further comparative studies between adolescent or juvenile TBIs and adult TBIs and (2) a specific approach adapted to adolescent/juvenile care in preclinical and clinical research.

### 2.3. Aged

TBI prevalence is high in aged populations and is associated with increased mortality and poorer functional outcomes in older patients. It is known that the microglial activation profile changes in the aged brain [197], but the underlying molecular and cellular mechanisms of secondary injury in the aged brain are still poorly understood, and aged TBI models are underrepresented in the literature [198]. Despite the small number of studies concerning it, they show the advantage to often compare to young adults, aged 3 months. Only rodents have been studied in the preclinical studies on aged animals. Rodents aged 12 to 18 months are assumed to correspond to middle-aged humans, and rodents aged 22 to 24 months are assumed to correspond to aged humans (Figure 1, Table 4 and Table 5).

#### 2.3.1. Post-Traumatic Inflammatory Consequences 

Published data on post-traumatic inflammatory consequences on aged animals are summarized in Table 4.

Microglia

Microglial activation induced by TBI in aged rodents occurs from 1 to 60 dpi and follow the same time/activation pattern than in young adults, but is exacerbated in older animals [199,200,202,206,207,209,210]. This could be explained by an alteration of microglia in aged brains, both quantitatively and qualitatively. Indeed, studies reported either a decrease [200] or an increase [206] in the absolute basal number of microglia in aged brains. Age also increases the expression of senescence markers in resident microglia, increasing their sensitivity to TBI and altering their proliferation potentials, phagocytic behavior and cytokinic profiles [200]. Moreover, microglial and infiltrating monocyte phagocytic capacities are impaired in aged brains compared with those of young adults, suggesting impairments regarding debris clearance mechanisms and the phagocytic removal of damaged cells in immune cells from aged rodents [200].

The different microglial phenotypes have not been deeply studied, but at 1 dpi, the M1 phenotype and most of the M2a phenotype genes are highly upregulated compared with young adults, in contrast with the M2c phenotype genes, which are downregulated [210]. Another study reported the reduced expression of M2a genes in aged TBI at 1 dpi compared with young adults, but this ratio was reversed at 5 dpi [205]. 

In the absence of any longitudinal study of phenotype-gene expression, it is difficult to establish a precise profile of microglial activation, but all these data highlight a different microglial activation profile in aged brains compared with young adults.

Astrocytes

As concerns microglia, astrocyte activation following TBI differs in aged compared with young adult populations. Astrocyte activation peaks and decreases earlier in young adult brains [201,206]. In aged brains, astrocyte activation is higher and persists longer, up to 60 d [199,206,207]. Moreover, a study reported the presence of degenerative astrocytes and the decrease of endfoot integrity following TBI in aged mice, but not in young adults [201]. Also, gene expression profile and astrocyte activation phenotype are different in aged TBI [201]. This implies that the astrocytic response after injury is altered both quantitatively and qualitatively in aged brains as compared with young brains.

Other immune cells’ and cytokines’ expression

The leukocyte infiltration process is also altered qualitatively and quantitatively. Qualitatively, more infiltration by neutrophils and fewer by monocytes was observed [200]. Quantitatively, the recruitment process in aged mice is emphasized and subchronically exacerbated as more newly recruited leukocytes are present in their brains than in those of young adults from 1 to 7 dpi [200,203,205,209]. However, a study reported altered neuroimmune response with fewer leukocytes infiltrating myeloid cells and microglia at 1 and 7 dpi in aged TBI rats, compared with young adults [211]. One explanation could stand on the dysregulation of chemokines’ expression, observed from 1 dpi in aged mice, with a decrease of anti-inflammatory chemokine CRX3CL1 and an upregulation of other chemokines’ expression between 4 and 7 dpi [209]. Moreover, after 7 dpi, the expression of most chemokines was unchanged, in contrast with young adults, whose expression of the same decreased [203].

Cytokine production is also different in aged mice. The baseline expression of some pro-inflammatory cytokines, such as IL-1β, TNFα, iNOS and IL-6, and chemokines, like MCP-1, are already higher in aged animals compared with young adults [205], indicating a subclinical chronic inflammatory process in the elderly, referred to as “inflamm-aging” [204]. The overall pattern of inflammatory protein expression differs between aged and young adults. Specifically, IL-1β and TNFα expressions peak earlier [204] and IL-6 expression decreases earlier [203] in aged animals. At 7 dpi, the expression of pro-inflammatory markers is similar between young adult and aged animals, but anti-inflammatory markers appear reduced in aged animals [203].

Interestingly, although overall cytokine expression is increased in aged animals, the production thereof seemed to be decreased in infiltrated leukocytes [200].

#### 2.3.2. Post-Traumatic Neuronal, Tissue and Functional Consequences

Published data on post-traumatic neuronal, tissue and functional consequences on aged animals are summarized in Table 5.

**Table 5:** Post-traumatic neuronal, tissue and functional consequences on aged animals. *Abbreviations:* *↑: increase; ↓: decrease; BBB: blood-brain barrier; CCI: controlled cortical impact; d: day post-injury; FP: fluid percussion; h: hour post-injury; LFP: lateral fluid percussion; M: post-natal month; NC: not compared; TBI: traumatic brain injury; W: post-natal week; YA: young adult*.

Animal	Age (Aged)	Comparison Age (YA)	Main Highlights		Reference
			Neuronal Death and Brain Lesion	Functional Deficits and Other Consequences	
**Mice** **LFP, open skull, unilateral**	M12	M1.5/3	- axonal degeneration aged > YA at 28 d - epothilone D ↑ axonal degeneration in aged at 28 d		[199]
**Mice** **CCI, open skull, unilateral**	M18	M3		- ↓ motor coordination aged > YA from 1 to 3 d - weight loss in aged from 2 d	[200]
**Mice** **CCI, open skull, unilateral**	M18	M3	- white mater injuries aged < YA at 30 d - ventriculomegaly aged < YA at 30 d	- inhibition of anxiety-like behavior aged > YA at 31 d - exploration deficit aged > YA at 67 d - learning and memory deficits aged > YA at 32 and 93 d	[213]
**Mice** **CCI, open skull, unilateral**	M19	NC	- lesion size aged > previous adult studies from 1 to 28 d - ↓ synapse at 30 d (hippocampus)		[202]
**Mice** **CCI, open skull, unilateral**	M20/25	M3/6		- depletion of monocytes infiltration = ↓ spatial memory deficits in aged at 30 d	[203]
**Mice** **CCI, open skull, unilateral**	M21	M2	- neuronal death aged > YA at 3 d	- neurological deficits aged > YA at 3 d - mortality rate aged > YA within 3 d	[204]
**Mice** **CCI, open skull, unilateral**	M21	M2		- neurological deficits aged > YA from 1 to 3 d - mortality rate aged > YA within 5 d	[205]
**Mice** **CCI, open skull, unilateral**	M21/24	NC	- ↓ neuronal density at 60 d (thalamus)		[207]
**Mice** **CCI, open skull, unilateral**	M21/24	M5/6	- neuronal death aged > YA at 3 d - no ≠ in lesion volume at 3 d	- locomotor deficits aged > YA at 14 d	[214]
**Mice** **LFP, open skull, unilateral**	M22/25	M5/6		- mortality aged > YA within 1 d	[208]
**Mice** **CCI, open skull, unilateral**	M24	M3	- antioxydant enzymes expression aged < YA at 1 d - lesion volume aged > YA at 7 d		[210]
**Rats** **LFP, open skull, unilateral**	M12	W10		- sensorimotor deficits M12 > W10 from 2 to 6 d	[211]
**Rats** **CCI, open skull, central**	M12/22	M3	- antioxidant capacities aged < YA at 1 and 7 d (cortex) - oxidative damage aged > YA at 1 and 7 d (hippocampus) - tissue loss aged > YA at 1 and 7 d		[215]
**Rats** **CCI, open skull, unilateral**	M14	NC	- no effect of nicotinamide on lesion size	- sensorimotor and memory deficits from 2 to 22 d - no effect of nicotinamide on functional deficits - no effect of nicotinamide on BBB leakage	[216]
**Rats** **CCI, open skull, centered**	M14	M3	- lesion volume aged > YA at 37 d	- motor deficit aged > YA at 25 d - memory deficit aged > YA at 5 d	[217]
**Rats** **FP, open skull, centered**	M20	M3		- motor and memory deficits aged > YA at 3 and 5 d	[218]
**Rats** **CCI, open skull, unilateral**	M20/22	NC		- sensorimotor deficits from 1 to 14 d - taurine: no effect on sensorimotor deficit or tissue loss (≠ with previous studies on younger animals)	[219]
**Rats** **Penetrating TBI, open skull, unilateral**	M20/22	M5/12	- lesion volume aged > YA at 1 d		[212]
**Rats** **CCI, open skull, unilateral**	M24	W10	- altered antioxidant capacities aged > YA at 7 d - neuronal death aged > YA at 7 d (cortex) - brain lesions aged > YA at 7 d	- learning and memory deficits in aged from 1 to 5 d, not in YA	[220]

Antioxidant response

In humans, brains’ antioxidant capacity decreases with age. Thus, some studies have shown interest in the antioxidant response in aged rodents. Following TBI, these animals show increased free radical production and lipid peroxidation [129,215,220]. Their overall antioxidant enzyme capacities are reduced at 1 and 7 dpi [129,210,215] and thus overwhelmed, resulting in a stronger oxidative stress response [220]. This stronger oxidative stress response might be one of the explanations for the exacerbated alteration of microglial activation and neuronal death seen at this age.

Neuronal death

Neurodegeneration and neuronal death are exacerbated at 3 and 7 dpi in the damaged area and hippocampus of aged mice, as compared with young adults [204,210,214,220]. Aged mice also showed more important WMI than young adults [213]. The evolution of axonal injuries differs by age, with early injury at 7 dpi in younger animals resolved by 28 dpi, whereas, in older animals, axonal injuries are less severe at 7 dpi but worsen by 28 dpi [199]. TBI also induces a decrease of synapse number in aged mice [203]. 

Lesion size

From 1 to 28 dpi, lesion volume is higher in aged mice than in young adults [203,210,212,220]. However, these results are inconsistent, especially at 3 dpi [204,205,214], as the cortical thickness and axonal degeneration are not systematically altered in aged mice [199].

Functional deficits

Age-related deficits in neurobehavioral functions consecutive to TBI are associated with weight loss and cognitive and behavioral deficits [200]. Indeed, TBI induces deficits in learning and memory, locomotor and motor coordination and anxiety-like behaviors [200,202,214,217,220]. Compared with young adults, aged mice show more important sensori- and locomotor [200,211,214,217,218], anxiety-like, exploratory behavior [213] and learning and memory deficits [213,217,218,220]. These neurobehavioral deficits persist up to 3 mpi [213].

Mortality rate

Globally, mortality is exacerbated in aged mice compared with young adults, as there is a higher death rate after TBI, from +18% at 1 dpi to +22% at 3 dpi for aged rodent compared with young adults [204,205,208].

#### 2.3.3. Anti-Inflammatory and Neuroprotective Therapeutic Strategies

The response to therapeutic strategies following TBI is not the same in aged TBI models compared with young adults.

Following TBI, inhibition of monocyte/macrophage infiltration in aged mice prevents spatial memory deficits [203] to a greater extent than in young adult mice [104].

Conversely, some neuroprotective strategies, such as the administration of taurine, an endogenous amino acid with antioxidant, anti-inflammatory and anti-apoptotic effects, and which is neuroprotective in adult TBI [221], is not effective following TBI in aged animals [219]. Moreover, nicotinamide, which improves functional outcome following young adult TBI [196], does not enhance functional recovery at low doses and even worsens functional deficit with higher doses [216]. The same phenomenon is observed with epothilone D, a cytoskeletal stabilizing agent used as an axonal protection therapy following TBI in young adult [222], which is actually detrimental to axonal integrity following TBI in aged animals [199]. 

These studies confirm that the response to different neuroprotective strategies varies with age. Thus, in addition to studying variation in consequences in terms of neuroinflammation, neuronal loss and functional deficits, the response to novel therapies must be studied in each age category.

#### 2.3.4. Conclusions

The investigation of the posttraumatic neuroinflammation pathway and its consequences in aged animals has just begun. Aged mice show altered basal inflammatory status, with higher levels of pro-inflammatory cytokines, senescence markers in their resident microglia and impairment of immune cells’ phagocytic capacities, compared with young adults [200]. This could explain the different inflammatory process in aged mice, especially their cytokine expression and glial activation [199,200,202,206,207,209,210]. This higher microglial activation may induce a vulnerability to secondary insults and promote psychological stress, and may, itself, exacerbate microglial activation [223]. Also, aged animals show poorer antioxidant capacities [129,210,215], poorer functional reserves and a decrease in neuronal plasticity [204]. This results in greater predisposition to neuronal loss and, thus, to loss of function, dementia-like symptoms and neurodegenerative pathologies [129]. It suggests that, following TBI, the aged brain is more reactive to neuroinflammation than the adult brain, and more vulnerable to neurodegeneration. This could explain the greater functional deficits and higher mortality rate, following TBI, in aged rodents, as compared with adults [200,202,204,205,208,211,213,214,217,218]

Administration of anti-inflammatory or neuroprotective components, like nicotinamide, taurine or epothilone, revealed no effect or even worsened tissue loss and functional deficit in aged TBI mice [199,216,219]. Altogether, these studies illustrate the complexity of age-dependent mechanism of the consequences of TBI and the necessity to consider its singularity in treatments of aged populations. 

Finally, all these studies assessed the consequences of TBI on “healthy” aged animals, which does not reflect the clinical reality, where elderly populations often suffer from other diseases like hypertension, kidney failure or neurodegenerative disease, that could alter neuroinflammation response even more [224,225,226]. Notably, a repetitive TBI model induced worse neurobehavioral impairments in an Alzheimer’s disease mice model than in a “healthy”-aged models [227].

## 3. Conclusions

TBI is most prevalent disease worldwide and can have the most devastating consequences in newborns, juvenile/adolescent and aged animals, due to the characteristics of developing or senescent brains. Yet, today, few preclinical studies have focused on post-traumatic neuroinflammation and its consequences at these ages. In this review, we have shown that the inflammatory brain response to and the neurological consequences of TBI vary with age. Indeed, immature or senescent brains do not show the same immune cell reactivity, antioxidant capacities or neuronal plasticity. For example, antioxidant enzyme capacities are diminished in aged rodents and the effect of stimulating GABA receptors is reversed in neonate rodents. These factors could help to explain the difference in the effects of strategies that are usually neuroprotective in young adults following TBI, but which are ineffective or even deleterious in neonates, juvenile, adolescent or aged animals (Table 6). Indeed, this review shows that, in neonates, strategies that are usually neuroprotective in young adults (administration of minocycline, cromoglycate), have been shown to be not or only transiently effective. The same phenomenon is observed in aged animals, with molecules that have demonstrated neuroprotective properties in young adults (taurine, nicotinamide, epothilone D) having no effect or even worsening neuronal loss. Interestingly, despite inconsistent results, the therapeutic strategies that are effective in adults seem to be equally effective in adolescents/juveniles. These studies confirm the need for an age-specific approach to developing neuroprotective strategies for the prevention and treatment of the consequences of TBI. Thus, the most effective research strategy might be to identify lesion mechanisms by age in order to develop therapeutic strategies adapted to each age, rather than extrapolating the results obtained in adults to other ages. 

Additionally, we currently lack direct comparative studies between ages. This is due to the difficulty in comparing the consequences of TBI between any given two ages using the same impact parameters. Brain size and plasticity vary depending on age, so the same impact parameters may correspond to different TBI severity, depending on age. It might be necessary to adapt impact parameters according to age, but this is necessarily done approximatively and empirically. Moreover, it is tricky to transpose these results to humans, due to the differences between rodent and human brains. Firstly, most preclinical studies are conducted on rodents whose brains are divided into two hemispheres, unlike humans. Most of the time, TBI is performed on a single hemisphere, which cannot be the case in humans. This can induce a variation in the induced cellular and molecular responses to TBI. Secondly, the classification of rodents according to their age is based on the chronology of brain development. This evolution may follow a different temporality if it is based on different criteria, such as metabolism or immunity. 

Finally, almost all the studies conducted at ages other than adult have been conducted in male animals. It is known that the consequences, in term of neuroinflammation, neuronal death and functional deficits, vary by sex [228,229]. For example, following TBI in adolescence, sex-dependent alterations in executive function are seen in early adulthood [228]. Thus, studies of the consequences of TBI on females of extreme age are also needed to complete our knowledge of TBI.

**Table 6:** Effect of neuroprotective strategies in different age groups. *Abbreviations:* *↑: increase; ↓: decrease; CCI: controlled cortical impact; CCR2: C-C chemokine receptor type 2; d: days post-injury; LFP: lateral fluid percussion; M: post-natal months; P: post-natal days; TBI: traumatic brain injury; WD: weight drop*.

Animal and TBI Model	Age	Molecule or Strategy	Effect on Post-Traumatic Inflammatory, Neuronal and Functional Consequences	Reference	Effects Observed in Adult TBI
**Neonates**					
Mice WD, closed head, unilateral	P7	minocycline	- ↓ microglia number at 1 d - ↓ ventricular dilatation and neuronal loss at 1 d - no effect at 5 d	[144]	- ↓ microglial activation [194] - ↓ lesion size [194] - ↓ functional deficits [194]
Mice WD, closed head, unilateral	P7	cromoglycate	- ↓ microglial activation markers at 1 and 5 d - no effect on microglial number at 1 and 5 d - no effect on neuronal and tissue loss at 1 and 5 d	[143]	- ↓ infiltrating mast cells degranulation [155] - ↓ microglial activation [230]
**Adolescent/juvenile**					
Rats CCI, open skull, unilateral	P17	docosahexaenoic acid	- ↓ brain atrophy and white matter injury at 3 and 12 d - ↓ acquisition deficits at 40 d	[173]	- ↓ microglial and astrocytic activation [231] - ↓ neuronal loss [193] - ↓ lesion size [231] - ↓ functional deficits [231]
Rats CCI, open skull, unilateral	P17		- ↓ microglial and astrocytic activation at 3 and 7 d - ↓ memory deficits at 14 d	[175]	
Rats CCI, open skull, unilateral	P17	minocycline	- ↓ microglial activation at 7 d - ↓ neuronal loss at 7 and 14 d - delays motor recovery at 5 d - ↓ memory deficits at 14 d	[174]	- ↓ microglial activation [194] - ↓ lesion size [194] - ↓ functional deficits [194]
Mice CCI, open skull, unilateral	P42	dexamethasone	- ↓ microglial and astrocytic activation from 1 to 14 d - ↓ peripheral immune cell recruitment - ↓ cognitive and behavioral deficits	[165]	- ↓ microglial activation [232] - ↓ apoptosis [233] - ↓ lesion size [233]
Mice CCI, closed head, unilateral	P35	isoflurane	- ↓ microglial activation at 1 d - ↓ axonal injury at 1 d	[164]	- ↓ neuronal loss [195] - ↓ cognitive deficits [195]
Rats CCI, open skull, unilateral	P28	nicotinamide	- ↓ microglial activation at 3 and 7 d - ↓ cortical tissue loss only at 3 d, not at 7 and 30 d - no effect on acquisition, locomotor and sensorimotor deficits at 30 d	[179]	- ↓ microglial activation [234] - ↓ lesion size [196] - ↓ behavioral deficits [234]
**Aged**					
Mice CCI, open skull, unilateral	M20/25	CCR2 knockout mice (macrophage infiltration inhibition)	- ↓ spatial memory deficits aged > young adult at 30 d	[203]	- ↓ cytokines expression [235] - ↓ neuronal loss [235] - ↓ locomotor and learning deficits [235]
Rats CCI, open skull, unilateral	M20/22	taurine	- No effect on tissue loss - No effect on sensorimotor deficits	[219]	- ↓ astrocytic activation [221] - ↓ pro-inflammatory cytokines [221] - ↓ neuronal loss [236] - ↓ functional deficits [221]
Rats CCI, open skull, unilateral	M14	nicotinamide		[216]	- ↓ microglial activation [234] - ↓ lesion size [196] - ↓ behavioural deficits [234]
Mice LFP, open skull, unilateral	M12	epothilone D		[199]	- ↓ axonal injury [222] - ↓ functional deficits [237]

In conclusion, most of the preclinical studies evaluating the consequences of TBI are conducted on young male adults. This does not reflect the clinical reality, and we question it, since our review highlights the variability of response by age. The complexity of performing TBI research on extreme ages and in comparing between different models leaves the consequences of TBI difficult to study. However, researchers should not give up, as it is crucial to know, precisely, all the mechanisms underlying the consequences of TBI in order to develop effective neuroprotective strategies.

## Figures and Tables

**Table 1:** Post-traumatic inflammatory, neuronal and functional consequences in neonatal animals. *Abbreviations: ↑: increase; ↓: decrease; CCI: controlled cortical impact; d: days post-injury; h: hours post-injury; NC: not compared; NMDA: N-methyl-D-aspartate; P: postnatal days; TBI: traumatic brain injury; WD: weight drop*.

Animal and TBI Model	Age	Compa-rison Age	Main Highlights						Reference
			Microglia	Astrocytes	Cytokines and Other Immune Cells	Brain Lesion and Neuronal Death	Functional Deficits	Comparison to Other Ages	
**Mice** **WD, closed head, unilateral**	P3/7	P10/14/30				- excitotoxic degeneration from 0.5 to 24 h; peak at 4 h (injury site) - apoptosis from 6 to 24 h (distant site)		- Brain injury P3/7 > P10/14/30	[140]
**Mice** **WD closed head, unilateral**	P7	NC				- neuronal death expansion from 0.5 to 6 h (edematous swelling of dendritic processes and acute edematous degeneration of neuronal cell bodies with pyknotic changes of the nuclei)			[141]
**Mice** **WD, closed head, unilateral**	P7	NC	- activation from 1 to 21 d	- activation from 5 to 21 d		- cellular death from 1 to 5 d - ventriculomegaly at 21 and 28 d			[142]
**Mice** **WD, closed head, unilateral**	P7	NC			- variation in pro- and anti-inflammatory cytokines/chemokines gene expression at 1 and 5 d - variation in pro- and anti-inflammatory immune cells gene expression at 1 and 5 d	- ventriculomegaly at 1 and 5 d			[143]
**Mice** **WD, closed head, unilateral**	P7	NC	- activation at 1 d - predominant reparatory/regenerative or immunomodulatory macrophage/microglia phenotype		- ↑ pro and anti-inflammatory cytokines expression from 1 to 5 d (< to adult) - microglia and macrophages depletion = transient ↓ pro-inflammatory cytokines	- thalamus, hippocampus and cortex tissue injury at 1 d - ventriculomegaly at 1 d- microglia and macrophages depletion = ↓ neuronal death and ventriculomegaly at 1 d			[144]
**Mice** **WD, closed head, unilateral**	P7	NC	- activation from 0.5 to 5 d			- excitotoxic degeneration at 1 d			[139]
**Mice** **WD, closed head, unilateral**	P7	NC				- ↑ neuronal mitochondrial activity and size at 1 d - ↓ 20% in sensorimotor cortical thickness at 30 d- Neuronal death still observed at 23 d			[145]
**Rats** **WD, closed head, unilateral**	P7	NC					- Neuronal death at 125 d (hippocampus) - Anxiety and spatial memory deficits at 125 d - NMDA antagonist improves morphological, histopathological, neurochemical and functional effects of brain injury at 125 d		[146]
**Rats** **WD, closed head, unilateral**	P3/7	P14/30				- cell death from 6 h to 5 d (peak at 24 h)		- apoptosis P3/P7 > P14/30	[147]
**Rats** **CCI, closed head, unilateral**	P11	NC				- ↑ intrinsic excitability and frequency of spontaneous excitatory post-synaptic currents at 28 d - ↓ frequency of spontaneous inhibitory post-synaptic currents at 28 d	- cognitive deficits at 28 d		[148]
**Rats** **CCI, closed head, unilateral**	P11	P17						- glial activation in P11, not in P17 at 3 d- brain atrophy and ventriculomegaly P11 > P17 at 14 and 28 d - acquisition and retention deficits P11 > P17 at 28 d	[149]
**Rats** **CCI, closed head, unilateral**	P11	NC	- activation at 3, 15 and 35 d (ipsilateral cortex)			- microglial depletion = ↑ neurodegeneration at 3 d	- microglial depletion = no spatial learning deficits at 28 d		[150]
**Rats** **CCI, closed head, unilateral**	P11	NC					- social recognition deficits, but intact sociability at 28 and 56 d - no memory deficit at 28 and 56 d		[151]

**Table 2:** Post-traumatic inflammatory consequences on juvenile and adolescent animals. *Abbreviations: ↑: increase; ↓: decrease; CCI: controlled cortical impact; CCL2: chemokine ligand 2; d: days post-injury; h: hours post-injury; HMGB1: high-mobility group box 1; IL: interleukin; LFP: lateral fluid percussion; NC: not compared; P: post-natal days; TBI: traumatic brain injury; TNFα: tumor necrosis factor α; W: post-natal weeks; WD: weight drop*.

Animal and TBI Model	Age	Compa-rison Age	Main Highlights				Reference
			Microglia	Astrocytes	Cytokines and Other Immune Cells	Comparison to Other Ages	
**Mice** **CCI, closed head, unilateral**	P17	NC		- activation 1, 7 and 30 d (corpus callosum)			[158]
**Mice** **CCI, closed head, unilateral**	P17	NC		- hypertrophic morphology and extension of processes from 1 to 30 d (ipsilateral cortex first, then spread in remote regions) - proliferation from 1 to 30 d (ipsilateral cortex first, then spread in remote regions)			[159]
**Mice** **CCI, closed head, unilateral**	P21	NC	- activation at 14 d				[160]
**Mice** **CCI, open skull, unilateral**	P21	W8/10				- microglial and astrocyte activation ↑ earlier and higher in P21 - ↑ in HMGB1 in P21 serum, not in adult - cellular inflammatory response varies with age	[161]
**Mice** **CCI, open skull, unilateral**	P21	NC	- activation from 3 to 35 d (corpus callosum)	- activation from 3 to 35 d (corpus callosum)			[162]
**Mice** **CCI, open skull, unilateral**	P21	NC	- no ↑ at 1 and 4 d - activation at 8 d (ipsilateral cortex)	- activation at 8 d (hippocampus)	- ↑ neutrophils at 4 d - ↑ lymphocytes T helper at 4 d		[163]
**Mice** **CCI, closed head, unilateral**	P35	NC	- activation at 1 d - isoflurane ↓ activation at 1 d	- no activation at 1 d (corpus callosum)			[164]
**Mice** **CCI, open skull, unilateral**	P42	NC	- activation from 1 to 14 d (cortex and nucleus accumbens) - dexamethasone ↓ activation	- activation from 1 to 14 d (cortex and nucleus accumbens) - dexamethasone ↓ activation	- dexamethasone ↓ peripheral immune cell recruitment		[165]
**Mice** **CCI, open skull, unilateral**	P42	NC	- activation at 1 and 30 d (cortex and nucleus accumbens)	- activation at 1 and 30 d (cortex and nucleus accumbens)	- ↑ inflammatory markers at 1 and 14 d - pro-inflammatory cytokines predominate at 14 d		[166]
**Mice** **CCI, open skull, unilateral**	P42	NC	- activation at 7 and 30 d (optical tract)	- activation at 7 and 30 d (optical tract)			[167]
**Mice** **CCI, open skull, unilateral**	P42	W8				- variation in infiltrated immune cell profile and ↑ microglial phagocytosis of neuronal proteins in P42 - duration of microglial activation and phagocytosis and monocytes infiltration P42 > W8	[168]
**Rabbits** **CCI, open skull, unilateral**	P5/7	NC	- activation at 7 d (ipsi- and contralateral white matter tracts)				[131]
**Rabbits** **CCI, open skull, unilateral**	P5/7	NC			- ↑ pro- and anti-inflammatory cytokine from 6 h to 21 d (peak at ≠ time points) - pro-inflammatory cytokines ↑ within the 6 h, peak between 6 h and 3 d, ↓ at 7 d and stay elevated at 21 d - anti-inflammatory cytokines peaked at 6 h and then ↓ quickly		[169]
**Rats** **WD, closed head, centered**	P17	NC		- activation from 1 to 7 d, peak at 3 d (hippocampus, white matter tracts, corpus callosum, brainstem and forebrain)			[170]
**Rats** **CCI, closed head, centered**	P17	NC		- activation from 8 d to 18 d			[171]
**Rats** **CCI, open skull, unilateral**	P17	NC		- ↑ *gfap* expression at 1 d and 4 d	- ↑ *ccl2* expression at 1 d and 4 d		[172]
**Rats** **CCI, open skull, unilateral**	P17	NC			- ↑ TNFα, IL-1β, CCL2, and IL-6 at 1 and 2 d		[173]
**Rats** **CCI, open skull, unilateral**	P17	NC	- activation at 7 d (ipsilateral cortex, hippocampus and thalamus) - minocycline ↓ activation				[174]
**Rats** **CCI, open skull, unilateral**	P17	NC	- activation at 3 and 7 d (cortex and hippocampus) - docosahexaenoic acid ↓ activation	- activation at 3 and 7 d (cortex) - docosahexaenoic acid ↓ activation			[175]
**Rats** **CCI, open skull, unilateral**	P21	NC	- activation at 21 d (ipsilateral hippocampus)				[176]
**Rats** **CCI, open skull, unilateral**	P21	NC			- ↑ IL-1β at 21 d (ipsilateral prefrontal cortex and hippocampus)		[177]
**Rats** **LFP, open skull, unilateral**	P24/31	NC		- activation at 2 d			[178]
**Rats** **CCI, open skull, unilateral**	P28	NC	- activation at 3 h, 3 d and 7 d, no more at 30 d (cortex, hippocampus and dorsal thalamus) - nicotinamide ↓ activation at 3 and 7 d (cortex, hippocampus and dorsal thalamus)				[179]
**Rats** **WD, closed head, centered**	P30	NC			- ↑ inflammatory markers at 1 d (plasma) and 3 d (plasma and cerebellum) - ↑ microglia/macrophage activation markers at 1 and 21 d, mostly due to peripheral infiltrating cells		[180]
**Rats** **CCI, closed head, unilateral**	P35	NC		- activation at 1 d			[138]

**Table 3:** Post-traumatic neuronal, tissue and functional consequences on juvenile and adolescent animals. *Abbreviations: ↑: increase; ↓: decrease; CCI: controlled cortical impact; d: day post-injury; h: hour post-injury; IL: interleukin; m: month post-injury; LFP: lateral fluid percussion; MBP: Myelin Basic Protein; P: post-natal day; TBI: traumatic brain injury; W: post-natal weeks; WD: weight drop*.

Animal and TBI Model	Age	Compa-rison Age	Main Highlights			Reference
			Neuronal Death and Brain Lesion	Functional Deficits	Comparison to Other Ages	
**Mice** **CCI, closed head, unilateral**	P17	NC	- no major axonal disruption at 1 d - white matter alteration at 7 d (corpus callosum)	- motor deficits at 30 d - memory deficits at 30 d - anxiety-like behavior at 30 d		[158]
**Mice** **CCI, open skull, unilateral**	P21	NC	- neuronal loss at 1 d (ipsilateral subcortical white matter), 3 d (ipsilateral external capsule, caudate putamen and contralateral subcortical white matter) and 7 d (internal capsule, pyramidal tracts, and cerebellar peduncles) - neuronal loss at 7 d is in regions where microglia/macrophage activation is more prominent (ipsilateral cortex, hippocampus, and thalamus)			[181]
**Mice** **CCI, open skull, unilateral**	P21	NC	- ↓ oligodendrocyte progenitor cells and post-mitotic oligodendrocytes at 35 d (corpus callosum) - myelin fragmentation at 35 d (external capsule) - tissue loss at 35 d (perilesional regions)	- anxiety-like behavior and social memory deficits at 35 d - no locomotor deficits at 35 d		[162]
**Mice** **CCI, open skull, unilateral**	P21	NC	- tissue loss at 8 d (cortex)			[163]
**Mice** **CCI, open skull, unilateral**	P21/25	W8/12			- mortality rate P21 > adult (mostly due to surgical complications) - motor performance impairment W8/12 > P21 but no other behavioral tests	[182]
**Mice** **CCI, closed head, unilateral**	P35	NC	- isoflurane ↓ axonal injury at 1 d			[164]
**Mice** **CCI, open skull, unilateral**	P42	NC			- dexamethasone ↓ cognitive and behavioral deficits	[165]
**Mice** **LFP, open skull, unilteral**	P42	NC	- tissue loss and neuronal death at 16 d	- acquisition deficit at 9 and 19 d - motor deficit from 1 to 16 d		[183]
**Mice** **CCI, open skull, unilateral**	P42	NC	- neuronal loss at 7 and 30 d (optic tract)	- optokinetic response and visual acuity deficit at 7 and 30 d - no circadian rhythm alteration at 7 and 30 d		[167]
**Mice** **CCI, open skull, unilateral**	P42	W8			- ↑ sensitivity to cocaine rewarding effects in P42 - no difference in the alteration of other behaviors - ↓ synaptic arborization complexity and spine density in the nucleus accumbens, and alteration of dopamine system–related genes expression P42 > W8	[168]
**Mice** **WD, closed head, central**	P42	W9			- hippocampal neurodegeneration only in P42, correlated to microglial activation (not astrocyte activation) - motor deficits recovery at 16 d in P42 = W9 - memory deficits at 3–4 d and 4–16 d only in P42 - mortality rate P42 > W9	[184]
**Rabbits** **CCI, open skull, unilateral**	P5/7	NC	- brain lesion at 3 and 7 d - brain atrophy at 21 d (ipsilateral hemisphere)	- cognitive deficits at 9 and 19 d		[131]
**Rats** **WD, closed head, centered**	P17	NC	- axonal injury from 3 to 7 d correlated with astrogliosis localization (brainstem and forebrain)			[170]
**Rats** **CCI, closed head, centered**	P17	NC	- axonal injury at 6 h, 3 d, 8 d and not at 18 d, correlated with astrogliosis localization	- acquisition and memory deficits from at 4–7 d and 14–17 d		[171]
**Rats** **CCI, open skull, unilateral**	P17	NC	- loss of parietal neurons at 60 d - MBP fragmentation ↑ at 3 d, normal at 7 and 30 d, and ↑ at 60 d consistently to axonal conductance decrease - brain lesion ↓ from 3 to 60 d - cortical atrophy from 7 to 60 d	- sensorimotor deficits at 60 d - anxiety-like behavior at 60 d		[79]
**Rats** **CCI, open skull, unilateral**	P17	NC	- neuronal loss at 3 and 6 m (ipsilateral and contralateral hippocampus) - brain atrophy at 3 and 6 m (corpus callosum and cortex	- complex pattern of cognitive and motor deficits from 3 to 6 m		[185]
**Rats** **CCI, open skull, unilateral**	P17	NC			- Sensorimotor deficits from 1 to 7 d	[172]
**Rats** **CCI, open skull, unilateral**	P17	NC	- brain atrophy at 3 and 50 d - white matter injury at 12 d (corpus callosum) - docosahexaenoic acid ↓ brain atrophy and white matter injury	- acquisition deficits at 40 d - docosahexaenoic acid ↓ acquisition deficits		[173]
**Rats** **CCI, open skull, unilateral**	P17	NC	- neuronal loss at 7 and 14 d (thalamus and hippocampus) - minocycline ↓ neuronal loss (only in thalamus at 7 d and in hippocampus and thalamus at 14 d)	- minocycline delays motor recovery in the 5 d - minocycline ↓ memory deficits at 14 d		[174]
**Rats** **CCI, open skull, unilateral**	P17	NC			- memory deficits at 14 d- docosahexaenoic acid ↓ memory deficits	[175]
**Rats** **CCI, open skull, unilateral**	P17	NC	- brain lesion, hemorrhage and edema ↑ from 6 h to 3 d, correlated with TBI severity - degenerating neurons at 3 d ↑ with TBI severity			[186]
**Rats** **LFP, open skull, unilateral**	P19	NC	- no neuronal loss at 14 d	- acquisition deficits at 14 d		[187]
**Rats** **CCI, open skull, unilateral**	P21	NC	- brain lesion at 14 d < 90 d - loss of neurons at 14 d < 90 d	- acquisition deficits at 14 d < 90 d - anxiety deficits at 14 d < 90 d		[188]
**Rats** **CCI, open skull, unilateral**	P21	NC		- normal social behavior between 14 and 21 d (adolescence) but impaired between 39 and 49 d (adulthood)		[189]
**Rats** **CCI, open skull, unilateral**	P21	NC		- no impact on cognitive deficits at 11 d		[176]
**Rats** **CCI, open skull, unilateral**	P21	NC		- learning deficits at 14–19 d, correlated to IL-1β expression (ipsilateral prefrontal cortex and hippocampus)		[177]
**Rats** **CCI, open skull, unilateral**	P21	NC	- white matter injury at 6 m			[190]
**Rats** **CCI, open skull, unilateral**	P28	NC	- cortical tissue loss at 3, 7 and 30 d - nicotinamide ↓ cortical tissue loss only at 3 d	- nicotinamide does not improve acquisition, locomotor and sensorimotor deficits at 30 d		[179]
**Rats** **WD, closed head, centered**	P30	NC		- anxiety like behavior at 3 d - memory deficits at 7 d		[180]
**Rats** **WD, closed head, central**	P35	NC		- motivation deficits at 19 d		[191]

**Table 4:** Post-traumatic inflammatory consequences on aged animals. *Abbreviations:* *↑: increase; ↓: decrease; CCI: controlled cortical impact; CD: cluster of differentiation; CX3CL1: chemokine (C-X3-C motif) ligand 1; d: days post-injury; h: hours post-injury; IFNγ: interferon γ; IL: interleukin; iNOS: inducible nitric oxide synthase; LFP: lateral fluid percussion; M: post-natal months; MCP-1: monocyte chemoattractant protein-1; NC: not compared; TBI: traumatic brain injury; TGFβ: transforming growth factor β; TNFα: tumor necrosis factor α; W: post-natal weeks; YA: young adult*.

Animal and TBI Model	Age (Aged)	Compa-rison Age (YA)	Main Highlights			Reference
			Microglia	Astrocytes	Cytokines and Other Immune Cells	
**Mice** **LFP, open skull, unilateral**	M12	M1.5/3	- activation aged >> YA at 7 and 28 d	- activation aged >> YA at 7 and 28 d		[199]
**Mice** **CCI, open skull, unilateral**	M18	M3	- number aged < YA at baseline and 3 d - ↑ aged > YA at 3 d - ↓ phagocytic capacities in aged at 3 d		- TNFα, and IL-1β expression aged > YA at 3 d - ↑ immune cells infiltration at 3 d	[200]
**Mice** **CCI, open skull, unilateral**	M18	M4		- activation in aged from 1 to 7 d (hippocampus and cortex) - Activation in YA from 1 to 3 d - Gene expression of reactive astrocytes, inflammatory response, complement pathway, and synaptic support are modified in aged - Loss of astrocytes function and astrogliopathy in aged - No clear dichotomy A1/A2 in aged		[201]
**Mice** **CCI, open skull, unilateral**	M19	NC	- ↑ at 7 and 30 d (hippocampus) - ↑ at 10 and 30 d (total brain) - ↑ phagocytic activity at 30 but not 10 d			[202]
**Mice** **CCI, open skull, unilateral**	M20/25	M3/6			- ↑ pro-inflammatory markers (IL-1β, TNFα, and iNOS) in aged at 7 d - ↓ anti-inflammatory markers (Ym1, CD206, TGFβ, and IL-4Ra) in aged at 7 d - chemotactic ligands expression aged > YA at 4 and 7 d - monocyte infiltration aged > YA at 4 and 7 d	[203]
**Mice** **CCI, open skull, unilateral**	M21	M2			- IL-1β, TNFα and IL-6 expression aged > YA at baseline - IL-1β expression in aged > YA at 3 d	[204]
**Mice** **CCI, open skull, unilateral**	M21	M2	- M2a phenotype aged > YA at 1 d - M2a phenotype aged < YA at 5 d		- inflammatory markers expression aged ≠ YA at 1 and 5 d	[205]
**Mice** **CCI, open skull, unilateral**	M21/24	M5/6	- number aged > YA at baseline and from 1 to 28 d (hippocampus)	- number aged > YA at baseline and from 1 to 28 d (hippocampus)		[206]
**Mice** **CCI, open skull, unilateral**	M21/24	NC	- ↑ at 30 and 60 d (thalamus)			[207]
**Mice** **LFP, open skull, unilateral**	M22/25	M5/6			- cytokine expression profile aged ≠ YA	[208]
**Mice** **CCI, open skull, unilateral**	M23	M3	- activation aged > YA at 1 d		- Anti-inflammatory chemokine CX3CL1 ↓ in aged at 1 d - recruitment and activation of peripheral immune cells aged > YA at 1 d	[209]
**Mice** **CCI, open skull, unilateral**	M24	M3	- activation aged > YA at 7 d (cortex and hippocampus) - M1 phenotype genes expression levels aged > YA at 1 d (cortex) - variation in M2a phenotype genes expression at 1 d (cortex) - M2c phenotype genes expression aged < YA at 1 d (cortex)			[210]
**Rats** **LFP, open skull, unilateral**	M12	W10			- number of leukocytes, myeloid cells and microglia aged < YA (ipsilateral hemisphere)	[211]
**Rats** **Penetrating TBI, open skull, unilateral**	M20/22	M5/12	- number aged < YA at 2 d	- number aged > YA at 2 d		[212]

## Abbreviations

BBB	blood-brain barrier
CNS	central nervous system
dpi	days post-injury
hpi	hours post-injury
M	post-natal months
MBP	myelin basic protein
mpi	month post-injury
P	post-natal days
TBI	traumatic brain injury
W	post-natal weeks
WMI	white matter injury
